# Serum Resistin Level and Its Receptor Gene Expression in Liver Biopsy as Predictors for the Severity of Nonalcoholic Fatty Liver Disease

**DOI:** 10.5005/jp-journals-10018-1102i

**Published:** 2014-07-28

**Authors:** Mona Hegazy, Soheir Abo-Elfadl, Abeer Mostafa, Magdy Ibrahim, Laila Rashed, Ahmed Salman

**Affiliations:** 1 Department of Internal Medicine, Faculty of Medicine, Cairo University, Giza, Egypt; 2 Department of Pathology, National Cancer Institute, Cairo, Giza, Egypt; 3 Department of Gynecology, Faculty of Medicine, Cairo University, Giza, Egypt; 4 Department of Biochemistry, Faculty of Medicine, Cairo University, Giza, Egypt

**Keywords:** NAFLD, Obesity, Resistin, Liver biopsy.

## Abstract

**Background:**

Liver histology remains the gold standard for assessing nonalcoholic fatty liver disease (NAFLD). Noninvasive serological markers have been developed to evaluate steatosis to avoid biopsy. In NAFLD patients, serum resistin was higher than those in control lean and obese patients.

**Objective of the study:**

To investigate serum resistin and its receptor gene expression in liver biopsy as predictors for NAFLD severity.

**Patients and methods:**

This study was conducted on 54 obese patients, with suspected fatty liver by ultrasound (excluding diabetic, alcoholic, hepatitis C virus antibody (HCVAb) or hepatitis B surface antigen (HBsAg) positive patients). They were subjected to anthropometric measurements, laboratory studies including serum resistin, abdominal ultrasonography (US) and liver biopsy. The 15 lean subjects were included as a control group. According to biopsy results, patients were subdivided into nonalcoholic steatohepatitis (NASH) group (46 patients) and non-NASH group (8 patients).

**Results:**

Significantly higher levels of resistin were detected in NAFLD patients compared to control subjects (p = 0.0001). Also, higher levels of resistin were recorded in NASH group compared to the non-NASH group; however, the difference was not statistically significant (p = 0.584). Serum alanine aspirate aminotransferase (AST), alanine aminotransferase (ALT) and gamma-glutamyl transpeptidase (GGT) were higher in NASH patients than non-NASH group (p = 0.223, p = 0.005 and p = 0.006 respectively). Abdominal US showed high sensitivity in NAFLD diagnosis (sensitivity of sonar in detecting steatosis grade compared to biopsy was 61% in grade 1, 25% in grade 2 and 75% in grade 3).

**Conclusion:**

Serum resistin can be combined with other noninvasive markers to predict the presence of NASH as an alternative to liver biopsy.

**How to cite this article:** Hegazy M, Abo-Elfadl S, Mostafa A, Ibrahim M, Rashed L, Salman A. Serum Resistin Level and Its Receptor Gene Expression in Liver Biopsy as Predictors for the Severity of Nonalcoholic Fatty Liver Disease. Euroasian J Hepato-Gastroenterol 2014;4(2):59-62.

## INTRODUCTION

Nonalcoholic fatty liver disease (NAFLD) is a manifestation of metabolic syndrome, a group of conditions including hypertension, high plasma glucose, excess body fat around waist or abnormal cholesterol levels, which will work together to increase the risk of heart disease, diabetes, and stroke.^1^ In obese and overweight individuals, NAFLD prevalence can be as high as 70%. The pathological spectrum of NAFLD ranges from simple hepatic steatosis to more severe manifestations, such as nonalcoholic steatohepatitis (NASH), hepatic fibrosis and cirrhosis.^2^ Liver histology remains the gold standard for assessing disease severity in NAFLD. Being invasive, biopsy is unsuitable for community studies and particularly for studying hepatic fibrosis progression and also histological assessment of NAFLD due to sampling error. This can lead to underestimation of the fibrosis score, especially when specimen is small.^3^ Noninvasive panels of serological markers have been developed to evaluate the presence of steatosis and hepatic necroinflammation to avoid liver biopsy.^4^

Resistin, an adipocyte-derived signaling polypep-tide, was originally identified in 2001.^5^ Increased serum resistin levels are associated with metabolic syndrome and insulin resistance.^6^ In patients with NAFLD, serum resistin levels were higher than those in control lean and obese patients.^7^ This study was done to investigate serum resistin level and its receptor gene expression in liver biopsy as predictors for the severity in NAFLD.

## PATIENTS AND METHODS

This study was performed in Kasr El Aini Hospital, Internal Medicine Outpatient Clinic, Egypt, over a period of 11 months (from December 2011-October 2012) on 54 obese subjects [body mass index (BMI) < 30 kg/m^2^)]. They attended the hospital with complains of dyspepsia (32%), osteoarthritis (28%), back pain (22%) and obesity (18%).

The selection of participants in this study was based on inclusion criteria as follows:

- Age above 18 years
- BMI above 30 kg/m^2^, and US evidence of bright liver without or with hepatomegaly on abdominal ultrasound.
  The exclusion criteria included the following:
- Alcoholics
- Patients with chronic hepatitis B and C
- Diabetics

Each participant was subjected to provide full medical history, full clinical examination and anthropometric measurements [weight and height of each participant were measured, while the participant was clothed only in a light gown, and the BMI was calculated as body weight in kilograms divided by height square in meters (kg/m^2^), also waist circumference was measured midway between rib margin and the iliac crest in a standing position by the same examiner].

Serum biochemistry profile [including blood glucose level, serum total cholesterol, high-density lipoprotein (HDL)-cholesterol, low-density lipoprotein (LDL)-cholesterol, triglyceride levels, serum levels of ALT, AST and GGT)] was done for every participant.

Assay of serum resistin level was done using the quantitative sandwich enzyme immunoassay technique the kit supplied by Quantikine R&D System, USA. A monoclonal antibody specific for the resistin globular domain has been precoated onto a microplate. Standards and samples are pipetted into the wells and resistin present is bound by the immobilized antibody. After washing away any unbound substances, an enzyme-linked monoclonal antibody specific for the resistin globular domain is added to the wells. Following a wash to remove any unbound antibody-enzyme reagent, a substrate solution is added to the wells and color develops in proportion to the amount of resistin bound in the initial step. The color development is stopped and the intensity of the color is measured.^8^

Abdominal US was performed by the same operator (MH) using a Toshiba Aplio XV scanner equipped with a broad band 5.3 MHz curved array probe to assess the presence of liver steatosis (bright liver).

Liver biopsy was taken for all patients who were suspected to have NAFLD by abdominal US. Liver biopsy was fixed in ten percent neutral buffered formalin then embedded in paraffin blocks. Five micrometer thick sections were cut and stained with hematoxylin and eosin and examined under light microscope for histopatho-logical diagnosis and scoring using NAS scoring system according to Histological Scoring System for NAFLD.^9^ This scoring system addresses the full spectrum of lesions of NAFLD and allows a diagnostic categorization into NASH, borderline NASH or not NASH. Fibrosis staging was evaluated (separately from NASH) from 0 to 4 scales.^10^ As regarding statistical analysis, we considered borderline NASH and NASH as one group to facilitate comparison between the two studied groups (NASH and non-NASH).

### Detection of Resistin Gene Expression using Real-time PCR (RT-PCR)^11^

RNA extraction

Total RNA was isolated from liver tissue homogenates using RNeasy Purification Reagent (Qiagen, Valencia, CA) according to manufacturers’ instructions. The purity (A260/A280 ratio) and the concentration of RNA were obtained using spectrophotometry (Gene-Quant 1300, Uppsala, Sweden). RNA quality was confirmed by gel electrophoresis.

cDNA Synthesis

First-strand cDNA was synthesized from 4 μg of total RNA using an Oligo(dT)^12-17^ primer and Superscript™ II RNase Reverse Transcriptase. This mixture was incubated at 4°C for 1 hours, the kit was supplied by SuperScript Choice System (Life Technologies, Breda, The Netherlands).

Real-time Quantitative Polymerase Chain Reaction

Real-time polymerase chain reaction (RT-PCR) amplification was carried out using 10 μl amplification mixtures containing power SYBR Green PCR Master Mix (Applied Biosystems, Foster City, CA, USA), equivalent to 8 ng of reverse-transcribed RNA and 300 nM primers, the sequences of PCR primer pairs used for each gene are shown in Table 1. Reactions were run on an ABI PRISM 7900HT detection system (Applied Biosystems). PCR reactions consisting of 95°C for 10 minutes (1 cycle), 94°C for 15 s, and 60°C for 1 minute (40 cycles). Data were analyzed with the ABI Prism sequence detection system software and quantified using the vl-7, Sequence Detection Software from PE Biosystems (Foster City, CA). Relative expression of studied genes was calculated using the comparative threshold cycle method. All values were normalized to housekeeping gene glyceraldehyde-3-phosphate dehydrogenase (GAPDH).^12^

**Table 1:** **Table 1:** Primer sequences used for RT-PCR

*Primer*		*Sequence*	
Resistin		Forward: 5’ TCTAGCAAGACCCTGTGCTCCA 3’	
		Reverse: 5’ CTCAGGGCTGCACACGACAG 3’	
GAPDH		Forward: 5’ ACCACAGTCCATGCCATCAC 3’	
		Reverse: 5’ TCCACCACCATGTTGCTGTA 3’	

RT-PCR: Real-time polymerase chain reaction;

GAPDH: Glyceraldehyde-3-phosphate dehydrogenase

Fifteen healthy age matched non-obese subjects (BMI >25 kg/m^2^) with no manifestations of NAFLD by abdominal US were chosen as a control group.

## ETHICS

The study protocol conformed to ethical guidelines of the 1975 declaration of Helsinki, approved by Cairo University Research Ethics Committee (REC) (No. n-7-2011 in 28-5-2011). A written informed consent was obtained from all patients participating in the study.

## STATISTICAL METHODS

Data were statistically described in terms of mean ± standard deviation (±SD), median and range, or frequencies (number of cases) and percentages when appropriate. Comparison of numerical variables between the study groups was done using Kruskal-Wallis test with post-hoc multiple two-group comparisons. For comparing categorical data, Chi-square (χ^2^) test was performed. Exact test was used instead when the expected frequency is <5. Agreement between ultrasound and biopsy results was tested using kappa statistic. p-value <0.05 was considered statistically significant. All statistical calculations were done using computer programs SPSS (Statistical Package for the Social Science; SPSS Inc, Chicago, IL, USA) version 15 for Microsoft Windows.

## RESULTS

The present study includes 54 patients, suspected to have NAFLD (clinically and by abdominal US), and selected from the outpatient clinic. Also, it includes 15 age-matched nonobese healthy subjects as a control group. There were 50 females (92.6%) and four males (7.4%). The age of the participants ranged from 18 to 60 years (43.17 ± 7.37 years). BMI of the participants ranged from 30.2 to 42.1 kg/m^2^ (34.77 ± 3.76 kg/m^2^). The waist of the participants ranged from 81 to 146 cm (106.13 ± 14.99 cm).

Serum levels of resistin of the patients ranged from 1.05 ng/l to 9.8 ng/l (5.28 ± 2.39 ng/l, mean ± SD). Serum levels of resistin of the control subjects ranged from 1.2 ng/l to 3.02 ng/l (2.03 ± 0.64 ng/l). It was revealed that the levels of serum resistin were significantly higher in NAFLD patients compared to control subjects (p = 0.0001).

Resistin receptor gene expression in liver biopsy ranged from 0.1 to 0.79 with a mean ± SD of (0.3543 ± 0.24084).

According to the results of liver biopsy, patients were divided into two groups: group 1 includes patients who have NASH (NASH and borderline NASH by biopsy). They were 46 patients out of the 54 patients (85.1%). Group 2 includes patients who do not have NASH. They were only 8 patients out of the 54 patients (14.9%). There was no significant difference regarding sex, age, BMI, and waist (p > 0.05%) between these two groups (Table 2). Comparison between NASH and non-NASH groups as regards resistin levels revealed that serum levels of resistin and its receptor gene expression in liver biopsy were higher in NASH group than non-NASH group, but the difference was not statistically significant.

## DISCUSSION

The NAFLD represents a spectrum of disorders characterized by predominantly macrovesicular hepatic steatosis that occur in individuals in the absence of consumption of alcohol in amounts considered harmful to the liver.^13^ Measuring serum resistin, which is a protein hormone produced and secreted by adipocytes may serve as a predictor of progressive liver pathology in NAFLD.^14^ In the present study, serum resistin levels were significantly higher in NAFLD patients than control subjects (p = 0.0001). This agrees with the results of Pagano et al (2006) who found that resistin was higher in NAFLD patients compared to controls.^15^ Similarly, Jiang et al (2009) found that serum resistin concentrations were significantly increased in patients with NAFLD compared to control subjects.^14^

**Table 2:** **Table 2:** Comparison between NASH and non-NASH groups as regards anthropometric measurements

*Parameter*		*NASH*				*p-value*	
		*Yes*		*No*			
Sex: Male		2 (4.3%)		2 (25%)		0.1	
Female		44 (95.7%)		6 (75%)		0.1	
Age (year)		42.71 ± 4.519		45.75 ± 6.274		0.29	
BMI (kg/m^2^)		34.980 ± 3.952		33.588 ± 2.1912		0.643	
Waist (cm)		106.65 ± 15.534		103.13 ± 11.777		0.487	

According to the results of the liver biopsy, patients were divided into two groups: the first one included patients who were proven to have NASH (46 patients) and the second group included patients who do not have NASH (8 patients). In the present study, resistin level in blood was higher in the NASH group than the non-NASH group, but the difference was statistically insignificant.

This result partially agrees with Jiang et al who found that resistin concentrations directly correlated with the NASH score.^13^ Also, this partially agrees with the report of Younossi et al who found that histological NASH could be predicted by serum resistin.^16^

The current study found that resistin mRNA expression in liver biopsy was higher in NASH group than the non-NASH group, but this did not reach a statistical significance. This result also partially supports the data of Zhao et al who found that the expression of resistin mRNA increased in livers of NASH patients.^17^

Finally, it is worth to mention that there were some limitations in this study, such as low number of patients included in the study (many patients refused to do liver biopsy as they believe that there is no solid indication to do it), marked discrepancy of number of patients in both the groups (46 NASH patients *vs* 8 non-NASH patients), unequal sex distribution within the studied groups, and lower number of control subjects compared to studied patients.

Also, the presence of one pathologist in the study limited the value of the histology reads, but this was due to the limited number of patients included in the study, which made inclusion of additional number of pathologist difficult.
